# Influence of an inspiratory muscle fatigue protocol on older adults on respiratory muscle strength and heart rate variability. A randomized controlled trial

**DOI:** 10.3389/fnins.2024.1423927

**Published:** 2024-11-12

**Authors:** Arturo Ladriñán-Maestro, Jorge Sánchez-Infante, Daniel Martín-Vera, José Ángel Del-Blanco-Muñiz, Javier Merino-Andrés, Alberto Sánchez-Sierra

**Affiliations:** ^1^School for Doctoral Studies and Research, Universidad Europea de Madrid, Madrid, Spain; ^2^Research Group on Exercise Therapy and Functional Rehabilitation, Faculty of Sports Sciences, Universidad Europea de Madrid, Madrid, Spain; ^3^Faculty of Physiotherapy and Nursing of Toledo, Universidad de Castilla-La Mancha, Toledo, Spain; ^4^Faculty of Health Sciences, Universidad Francisco de Vitoria, Madrid, Spain; ^5^Physiotherapy Research Group of Toledo (GIFTO), Faculty of Physiotherapy and Nursing, Universidad de Castilla-La Mancha, Toledo, Spain; ^6^Faculty of Sport Sciences, Universidad Europea de Madrid, Madrid, Spain; ^7^Clínica Axium Salud Funcional, Madrid, Spain; ^8^Clínica Sierra Varona SL, Toledo, Spain; ^9^Department of Physiotherapy, Faculty of Health, Camilo José Cela University, Villanueva de la Cañada, Madrid, Spain; ^10^Department of Physical Therapy, Faculty of Health Sciences, Universidad Alfonso X El Sabio, Madrid, Spain

**Keywords:** older adults, autonomic cardiac control, heart rate variability, respiratory muscle strength, inspiratory muscle fatigue

## Abstract

**Introduction:**

Inspiratory muscle fatigue has been shown to have effects on the autonomic nervous system and physical condition. This study aimed to evaluate the influence of an inspiratory muscle fatigue protocol on respiratory muscle strength and heart rate variability in older adults.

**Materials and methods:**

A randomized controlled clinical trial with double-blinding was carried out involving 24 individuals over 60 years old who demonstrated physical independence in walking and movement. Participants were distributed randomly into three groups: Inspiratory muscle fatigue, activation and control. Measurements of heart rate variability, diaphragmatic ultrasound, and maximum inspiratory pressure were taken at two stages: prior to the intervention (T1) and directly after treatment (T2).

**Results:**

The inspiratory muscle fatigue group exhibited decrease scores in respiratory and heart rate variability subsequent to undergoing the diaphragmatic fatigue intervention compared to both the activation and control groups (*p* < 0.05). Conversely, the activation group demonstrated higher values in heart rate variability and respiratory capacity variables following the inspiratory muscle activation training (*p* < 0.05).

**Conclusions:**

Fatigue of the inspiratory musculature appears to negatively impact heart rate variability and inspiratory muscle strength in older adults.

**Clinical trial registration:**

https://clinicaltrials.gov/study/NCT06269042, identifier: NCT06269042.

## 1 Introduction

Aging is a natural and inevitable process that entails a series of negative physiological and cardiac changes, such as increased arterial stiffness, hypertrophy, alteration of diastolic function, or endothelial dysfunction (Lakatta and Levy, 2003), bearing in mind that in this age group, ~40% of deaths are due to cardiovascular diseases (North and Sinclair, 2012). Besides the intrinsic nervous system of the heart, there is a connection between the heart and the central nervous system (specifically the autonomic nervous system) to maintain proper homeostasis (Ritz et al., 2013), composed of afferent nerve fibers, interneurons, and efferent ganglia (the latter responsible for transmitting the signal through sympathetic and parasympathetic neurons (Tiwari et al., 2021).

Heart rate variability (HRV) is an intrinsic quality of the heart rate itself, defined as the variation between heartbeats over a specific period (Tiwari et al., 2021). The calculation of this variable can be carried out using linear methods (including time and frequency domains) or non-linear methods (Anonymous, 1996). In turn, this characteristic is mediated, among others, by the central nervous system, becoming an indirect measure of the sympathetic-parasympathetic relationship of the autonomic nervous system (Ernst, 2017; Cygankiewicz and Zareba, 2013), and can act as an indicator of the subject's intrinsic regulation capacity in response to various internal and external stimuli, as well as physiological and psychological stress (Arantes et al., 2022). Parasympathetic activity is decreased in older adults compared to healthy young individuals, and HRV is considered a valid biomarker of frailty in older adults (Arantes et al., 2022) showing correlations with stress (Thayer et al., 2010), cognitive function (Grassler et al., 2021), or cardiovascular diseases (Greiser et al., 2009).

The respiratory system, particularly the respiratory musculature, is closely related to the autonomic nervous system, especially through the diaphragm, due to its connection with the esophagus and the vagus nerve (Kocjan et al., 2017). Specifically, fatigue of the inspiratory musculature, through the metaboreflex, induces sympathetic hyperactivation mediated by afferent stimulation of type III and IV fibers (Romer and Polkey, 2008), as well as a redistribution of blood flow from active limbs to respiratory muscles (Sheel et al., 2018). There is evidence of improvements in HRV through unloaded breathing exercises (Laborde et al., 2022). Improvements in this variable have also been observed associated with loaded breathing exercises or inspiratory muscle training in both healthy individuals (Tanriverdi et al., 2021) and various clinical populations (Cutrim et al., 2019; Caruso et al., 2016). However, the available evidence regarding the effects of inspiratory muscle fatigue on HRV is very limited or non-existent.

Therefore, the objective of this study is to objectively evaluate the effects of an inspiratory fatigue protocol on inspiratory muscle strength and HRV in older adults.

## 2 Materials and methods

### 2.1 Study design

This research employed a randomized parallel clinical trial design and was conducted at the Physiotherapy Department of Residencial Montes de Toledo (Manzaneque, Spain), adhering to the Consolidated Standards of Reporting Trials (CONSORT) guidelines (Cuschieri, 2019). All participants provided informed consent. The study received approval from the Research Ethics Committee of the Complejo Hospitalario Universitario de Toledo (approval number: 1070) and was registered on ClinicalTrials.gov (NCT06269042).

### 2.2 Participants

Twenty-four older adults participated in the study and were randomly assigned using randomization.com software. Participants were categorized into three groups: the inspiratory muscle fatigue group (IMFG), control group (CG), and activation group (AG). An independent third party, unaffiliated with the study, conducted this allocation. Neither the evaluator nor the data analyst was aware of the group assignment for each participant. The inclusion criteria for participants in this study were: age over 60 years and independence in walking and transfers. Exclusion criteria encompassed individuals with cognitive impairments, tympanic perforation or middle-internal ear issues, pulmonary hypertension, decompensated cardiac or respiratory failure, those who had undergone lower extremity surgery in the past 12 months, refusal to sign the informed consent and participation in any specific exercise program for preventive or therapeutic purposes beyond the individual's own lifestyle or activity level. The sample size was calculated using G^*^Power Software (3.1.9.2), referencing maximal inspiratory pressure (MIP) data from a prior study (Holtzhausen et al., 2018). Parameters included an alpha error of 0.05, beta error of 0.2, and a medium effect size (*f* = 0.25 or Eta partial squared = 0.06). A 30% estimated dropout rate was factored in due to the study design. Consequently, a total of 24 participants were allocated, divided into three groups (*n* = 8) for the study.

### 2.3 Intervention

The Inspiratory Muscle Fatigue Group (IMFG) underwent an inspiratory muscle fatigue protocol using a threshold valve device (Big Breathe^®^ GH Innotek Co., Ltd., Busan, Republic of Korea). They breathed against submaximal inspiratory loads set at 60% of their maximal inspiratory pressure (MIP) until they failed to produce airflow in at least three maximal inspiratory efforts (Welch et al., 2018). The Activation Group (AG) adhered to a regimen of two sets of 30 repetitions at 40% of their MIP using the same threshold device as the IMFG, as referenced from another study (Ozdal, 2016). Conversely, the Control Group (CG) received no intervention. Participants in this group simply remained seated for the same duration as the intervention and activation groups needed to complete their protocol, which was ~10 min.

### 2.4 Outcomes

Two assessments were conducted at distinct time points: pre-intervention (T1) and immediately post-intervention (T2). The evaluator responsible for the measurements was unaware of the group assignments for each participant.

### 2.5 Primary outcomes

#### 2.5.1 Maximal inspiratory pressure

MIP was assessed using the MicroRPM^®^ Respiratory Pressure Measurement Device (MicroMedical, UK) with participants seated. To ensure mouth airflow, the nose was occluded. Subjects rested for 1 min between attempts and conducted up to six maneuvers. The highest consistent value from three efforts, with variability < 5%, was documented (Laveneziana et al., 2019).

#### 2.5.2 Diaphragmatic thickness and thickening fraction

Diaphragmatic thickness was assessed using a linear probe (L13-3s) operating at a frequency of 3.2–12.3 MHz, positioned perpendicularly to the chest wall in supine subjects. Measurements were taken at the anterior and mid-axillary lines, specifically between the 8th and 9th intercostal spaces. B-mode ultrasound was employed to visualize the diaphragm in the juxtaposition area. Thickness was measured thrice at both the end of expiration (Thick_exp_) and peak inspiration (Thick_insp_), with mean values recorded. The thickness at the end of expiration was defined as the diaphragmatic thickness. The thickening fraction (TF%) was computed using the formula: TF = [(Thickness at end of maximum inspiration – Thickness at end of expiration)/Thickness at end of expiration] × 100% (Santana et al., 2020).

#### 2.5.3 Diaphragmatic movement curve

**Figure 1:**
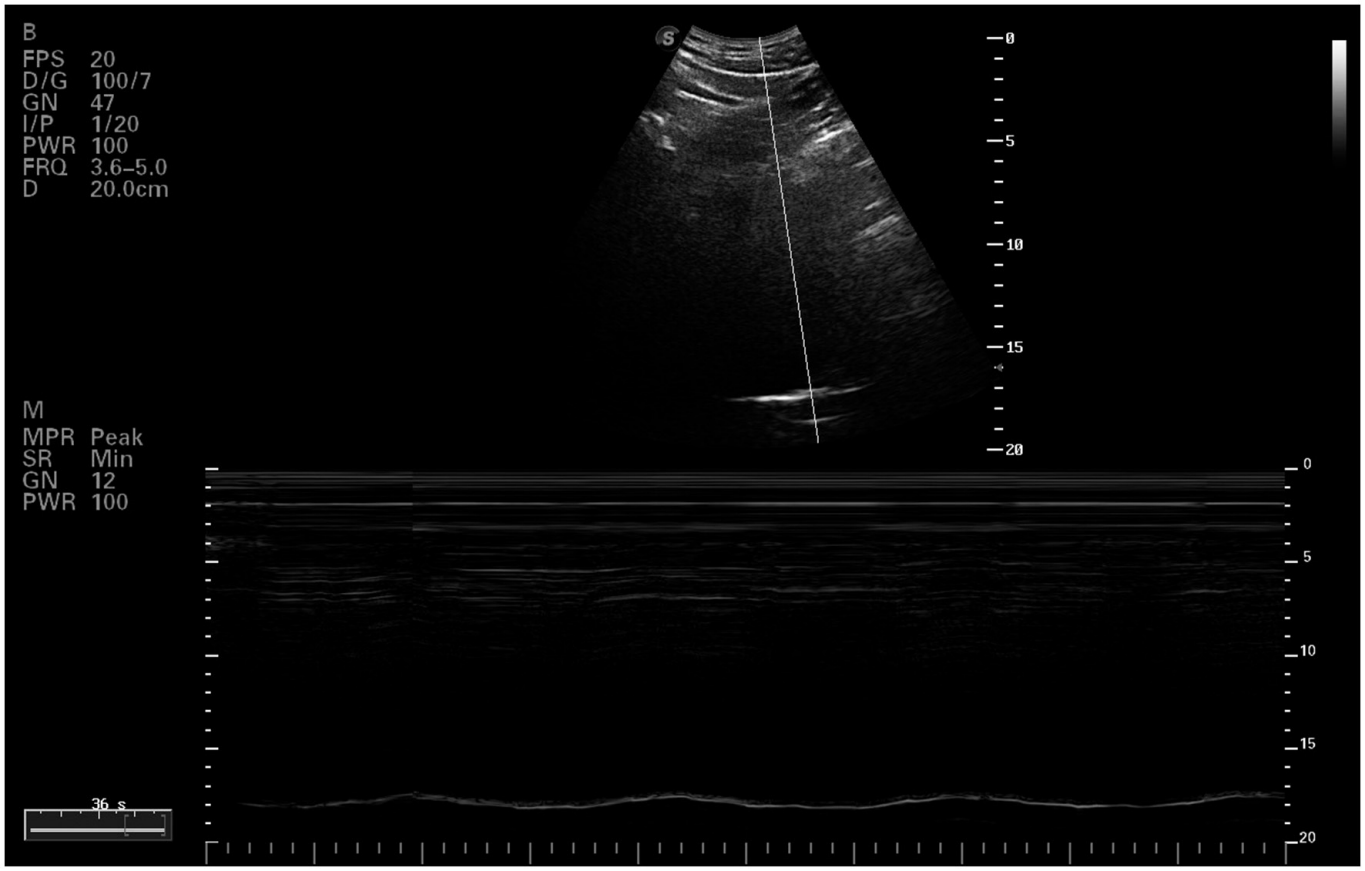
Ultrasound image of right diaphragmatic dome.

The evaluation of the diaphragmatic movement curve utilized a convex probe (C5-1s) operating at a frequency of 1.2–6 MHz. The probe was positioned longitudinally on the mid-clavicular line at the right costal margin, using the liver as an acoustic window. Subjects were in a supine position, and the probe was oriented cephalically. M-mode was employed to capture the diaphragmatic movement curve during both maximal deep breathing and sniff breathing (Figure 1). Parameters such as diaphragmatic excursion (Mob_insp_ and Mob_sniff_), inspiratory time (Time_insp_ and Time_sniff_), and maximum contraction velocity (Vel_insp_ and Vel_sniff_) were analyzed for each breathing type. Three consecutive respiratory cycles were assessed, and the average value for each parameter was recorded (Santana et al., 2020).

### 2.6 Secondary outcomes

#### 2.6.1 Heart rate variability

The analysis of HRV was conducted using a heart rate monitor (Polar H10; Polar Electro Oy, Kempele, Finland; Arantes et al., 2022). The cardiac electrical signals were monitored using a band placed on the chest for 5 min with the subject in a supine position on a stretcher, in a quiet environment, with soft lighting and a room temperature of ~25°C. Participants were asked not to consume caffeine, alcohol, tobacco, or engage in intense physical activity in the 12 h prior to the intervention. Additionally, they were instructed not to speak or make voluntary movements during the analysis (Tseng et al., 2020). The data were analyzed using Kubios HRV Analysis Software 3.1.0 for Windows (Biomedical Signal and Medical Imaging Analysis Group, Department of Applied Physics, University of Kuopio, Finland) and six parameters were determined: RR Interval (R-Ri), standard deviation of all normal-to-normal intervals (SDNN), power in low frequency (LF; 0.04–0.15 Hz) and power in high frequency (HF; 0.15–0.40 Hz), both presented in normalized units (nu), the sympathovagal balance index as the ratio between low and high frequency power (LF/HF), and the square root of the mean of the sum of squared differences between adjacent normal-to-normal intervals (RMSSD).

### 2.7 Statistical analysis

Statistical analysis was conducted utilizing IBM SPSS Statistics v.22.0. The significance threshold was established at *p* < 0.05. Normality of each variable was evaluated via the Kolmogorov-Smirnov test, indicating a normal distribution for all variables. Descriptive statistics were employed to scrutinize demographic characteristics, with measurements presented as mean ± SD. A 2-way repeated measures ANOVA was utilized for outcome variables, exploring the interaction among the Experimental group, Activation group, and Control group, and the time of assessment (Baseline, Posttreatment). *Post-hoc* Bonferroni multiple-comparisons tests were employed upon detection of differences. Effect size (ES) was interpreted according to Cohen's scale (Cohen, 1988): low (< 0.20), medium (0.50), and high (>0.80).

## 3 Results

### 3.1 Demographic data

**Figure 2:**
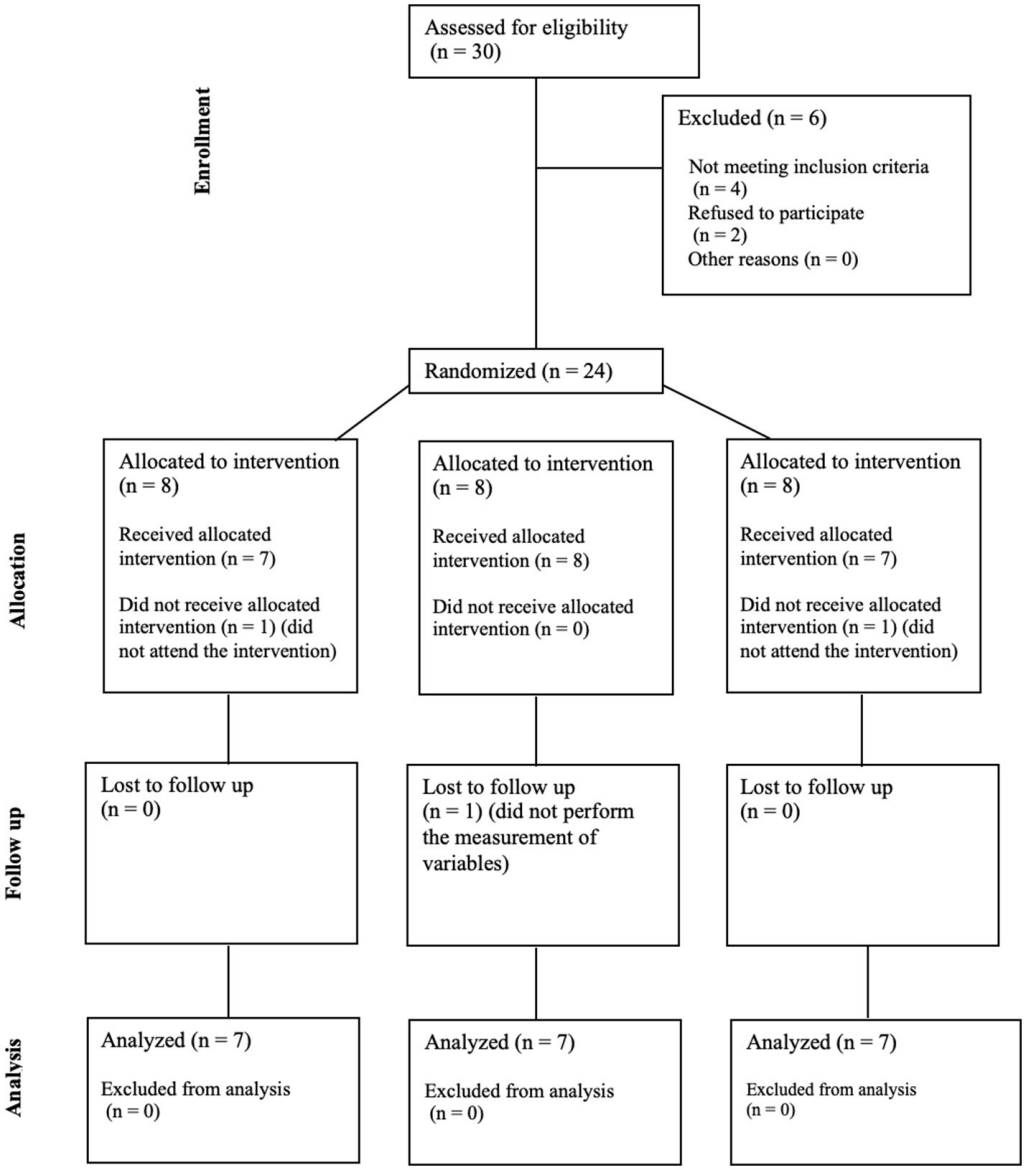
CONSORT flow chart.

Twenty-four older adults were recruited for the study on March 2024 and participate on April 2024. The participants did not take any new medications or any that could potentially interact at the cardiovascular level. They were distributed among IMFG (4-men, 3-women), AG (4-men, 4-women), and CG (5-men, 2-women). There were three dropouts due to the intervention and measurements. The CONSORT flow chart was included (Figure 2). No significant differences were found between IMFG, AG and CG in demographic characteristics (Table 1).

**Table 1:** Demographic characteristics of subject.

	**IMFG (*n* = 7)**	**AG (*n* = 7)**	**CG (*n* = 7)**	***p***
Sex (male/female)	4/3	4/4	5/2	
Age (yrs)	79.71 ± 7.34	78.71 ± 9.34	78.14 ± 9.06	n.s
Weight (kg)	66.71 ± 10.68	68.60 ± 15.07	69.67 ± 9.30	n.s
Height (cm)	171.14 ± 0.43	171.57 ± 8.02	170.71 ± 7.20	n.s
Physical Activity Level (minutes per week)	127.86 ± 44.33	117.14 ± 60.47	135.71 ± 51.92	n.s
First-Line Analgesics	2/7	3/7	3/7	
Vitamin D supplements	4/7	3/7	2/7	
Proton pump inhibitors	2/7	3/7	4/7	
Sedentary lifestyle	3/7	4/7	3/7	

IMFG, Inspiratory muscle fatigue group; AG, Activation group; CG, Control group.

### 3.2 Changes in respiratory variables

Results for primary outcomes are presented in Table 2.

**Table 2:** Outcome measurements of respiratory variables.

	**Baseline**	**Post-treatment**		***f***	***p***	***n* ^2^**	**Pot**
**MIP (cmH**_2_ **O)**							
IMFG	62.57 ± 10.10	55.43 ± 7.87^#**^	Group	1.24	0.31	0.12	0.24
AG	63.00 ± 6.48	66.00 ± 6.73^**^	Time	11.64	< 0.01	0.39	0.90
CG	64.29 ± 7.02	64.43 ± 6.10	Group × Time	59.72	< 0.01	0.87	1
**Thick**_insp_ **(cm)**							
IMFG	0.42 ± 0.09	0.39 ± 0.09^**^	Group	1.21	0.32	0.12	0.23
AG	0.46 ± 0.08	0.48 ± 0.08^**^	Time	13.07	< 0.01	0.42	0.93
CG	0.44 ± 0.07	0.44 ± 0.07	Group × Time	54.57	< 0.01	0.86	1
**Thick**_esp_ **(cm)**							
IMFG	0.23 ± 0.04	0.24 ± 0.04^**^	Group	0.11	0.90	0.01	0.06
AG	0.24 ± 0.05	0.23 ± 0.04	Time	1.04	0.32	0.06	0.16
CG	0.23 ± 0.03	0.23 ± 0.03	Group × Time	8.70	< 0.01	0.49	0.94
**TF (%)**							
IMFG	83.21 ± 18.09	62.37 ± 12.85^**##^	Group	13.01	< 0.01	0.59	0.99
AG	95.46 ± 7.48	106.26 ± 9.80^**^	Time	6.00	< 0.05	0.25	0.64
CG	96.18 ± 8.41	95.10 ± 8.76^++^	Group × Time	37.10	< 0.01	0.81	1
**Mob**_insp_ **(cm)**							
IMFG	5.45 ± 0.23	4.69 ± 0.24^**##^	Group	11.62	< 0.01	0.56	0.98
AG	5.52 ± 0.28	5.81 ± 0.33^**^	Time	8.38	< 0.05	0.32	0.78
CG	5.53 ± 0.26	5.52 ± 0.27^++^	Group × Time	32.12	< 0.01	0.78	1
**Time**_insp_ **(ms)**							
IMFG	1,971.43 ± 138.62	2,080.00 ± 145.60^##^	Group	3.68	0.04	0.29	0.60
AG	1,930.00 ± 136.14	1,770.00 ± 134.70^**^	Time	0.11	0.74	0.01	0.06
CG	2,010.00 ± 180.83	2,012.50 ± 118.05^$$^	Group × Time	3.21	0.06	0.26	0.54
**Vel**_insp_ **(cm/s)**							
IMFG	2.77 ± 0.13	2.32 ± 0.16^**##^	Group	15.56	< 0.01	0.63	1
AG	2.85 ± 0.23	3.22 ± 0.25^**$$^	Time	0.87	0.37	0.05	0.14
CG	2.76 ± 0.18	2.73 ± 0.19^++^	Group × Time	28.00	< 0.01	0.76	1
**Mob**_sniff_ **(cm)**							
IMFG	1.65 ± 0.19	1.54 ± 0.16^##^	Group	2.01	0.16	0.18	0.36
AG	1.64 ± 0.11	1.81 ± 0.07^*^	Time	0.01	0.91	0.00	0.05
CG	1.64 ± 0.19	1.57 ± 0.16^$$^	Group × Time	6.31	< 0.01	0.41	0.84
**Time**_sniff_ **(ms)**							
IMFG	162.86 ± 14.96	212.86 ± 16.04^**##^	Group	9.18	< 0.01	0.51	0.95
AG	165.71 ± 17.18	157.14 ± 17.99	Time	6.81	< 0.05	0.28	0.70
CG	162.86 ± 22.15	157.14 ± 13.80^++^	Group × Time	17.49	< 0.01	0.66	1
**Vel**_sniff_ **(cm/s)**							
IMFG	10.23 ± 0.60	7.18 ± 0.74^**++^	Group	15.25	< 0.01	0.63	1
AG	9.93 ± 0.57	11.60 ± 1.21^**##^	Time	13.61	< 0.01	0.43	0.94
CG	10.19 ± 0.73	10.04 ± 0.65^$^	Group × Time	97.69	< 0.01	0.92	1

IMFG, Inspiratory muscle fatigue group; AG, Activation group; CG, Control group; MIP, maximal inspiratory pressure; Thick_insp_, Diaphragmatic thickness in inspiration; Thick_esp_, Expiratory diaphragmatic thickness; TF, Thickness ratio inspiration/expiration; Mob_insp_, Maximal inspiration diaphragmatic mobility; Time_insp_, Maximum inspiratory contraction time; Vel_insp_, Maximum inspiration contraction velocity; Mob_sniff_, Diaphragmatic mobility sniff; Time_sniff_, Sniff contraction time; Vel_sniff_, Sniff contraction velocity.

Values are mean ± SD.

^*^P < 0.05, ^**^P < 0.01, post-treatment, with baseline.

^#^P < 0.05, ^##^P < 0.01, comparisons between the IMFG and AG groups at corresponding time points.

^+^P < 0.05, ^++^P < 0.01, comparisons between the IMFG and CG groups at corresponding time points.

^$^P < 0.05, ^$$^P < 0.01, comparisons between the AG and CG groups at corresponding time points.

In the analysis of the MIP variable, the IMFG had lower values than the AG after performing the treatment (*P* < 0.05). Within the IMFG analysis, there was a decreased between baseline and post-treatment of −7.14 ± 2.61 cmH_2_O (*p* < 0.01; ES = 0.71; 95% CI of the difference = −8.57 to −5.72). In contrast, the AG showed an increased between baseline and post-treatment of 3.00 ± 1.00 cmH_2_O (*p* < 0.01; ES = 0.46; 95% CI of the difference = 1.58 to 4.42).

In the analysis of the Thick_insp_ variable, the IMFG analysis, there was a decreased between baseline and post-treatment of −0.03 ± 0.01 cm (*p* < 0.01; ES = 0.37; 95% CI of the difference = −0.04 to −0.03). In contrast, the AG showed an increased between baseline and post-treatment of 0.01 ± 0.01 cm (*p* < 0.01; ES = 0.16; 95% CI of the difference = 0.00 to 0.02).

In the analysis of the Mob_sniff_ variable, the IMFG had higher values than the AG (*P* < 0.01), and the AG had higher values compared to the CG after performing the treatment (*P* < 0.01). Within the AG analysis, there was an increased between baseline and post-treatment of −0.76 ± 0.25 cm (*P* < 0.01; ES = 3.30; 95% CI of the difference = −0.96 to −0.56). In contrast, the AG showed an increased between baseline and post-treatment of 0.29 ± 0.19 cm (*P* < 0.01; ES = 1.05; 95% CI of the difference = 0.09 to 0.49).

### 3.3 Changes in heart rate variability

**Figure 3:**
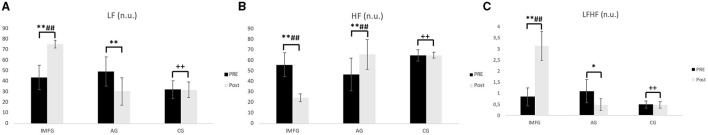
The frequency domain results. **(A)** LF, Low frequency; **(B)** HF, High frequency; **(C)** LFHF, sympathovagal balance index. Values are mean ± SD of pre and post-treatment. *P < 0.05, **P < 0.01, post-treatment, with baseline. ^#^*P* < 0.05, ^##^*P* < 0.01, comparisons between the IMFG and AG groups at corresponding time points. ^+^*P* < 0.05, ^++^*P* < 0.01, comparisons between the IMFG and CG groups at corresponding time points. ^$^*P* < 0.05, ^$$^*P* < 0.01, comparisons between the AG and CG groups at corresponding time points.

The time domain results are presented in Table 3. The frequency domain results can be seen in Figure 3.

**Table 3:** Outcome measurements of time domains of heart rate variability.

	**Baseline**	**Post-treatment**		***f***	***p***	***n* ^2^**	**Pot**
**HR (bpm)**							
IMFG	69.57 ± 9.78	79.00 ± 5.03^**#^	Group	0.85	0.44	0.09	0.17
AG	72.71 ± 5.96	66.00 ± 6.76^**^	Time	0.53	0.48	0.03	0.11
CG	70.00 ± 10.33	69.29 ± 9.74	Group × Time	26.50	< 0.01	0.75	1
**RR (ms)**							
IMFG	898.07 ± 121.70	702.51 ± 101.49^**##^	Group	1.11	0.35	0.11	0.22
AG	813.27 ± 101.42	893.28 ± 113.55^**^	Time	8.99	< 0.01	0.33	0.81
CG	890.45 ± 116.95	878.38 ± 146.75^++^	Group × Time	32.61	< 0.01	0.78	1
**SDDN (ms)**							
IMFG	50.42 ± 8.87	18.50 ± 3.35^**^	Group	1.16	0.34	0.11	0.23
AG	39.51 ± 32.12	50.80 ± 34.89^**^	Time	19.71	< 0.01	0.52	0.99
CG	53.01 ± 18.24	51.92 ± 19.41^+^	Group × Time	62.10	< 0.01	0.87	1
**RMSSD (ms)**							
IMFG	19.12 ± 7.36	12.46 ± 6.74^*##^	Group	0.18	0.84	0.02	0.07
AG	12.39 ± 7.40	23.03 ± 6.77^**^	Time	1.87	0.19	0.10	0.25
CG	15.71 ± 5.44	17.93 ± 5.27	Group × Time	10.46	< 0.01	0.57	0.97

IMFG, Inspiratory muscle fatigue group; AG, Activation group; CG, Control group; RR, RR Interval; SDDN standard deviation of all normal-to-normal intervals; RMSSD square root of the mean of the sum of squared differences between adjacent normal-to-normal intervals; HR, heart rate; BPM, beats per minute.

Values are mean ± SD.

^*^P < 0.05, ^**^P < 0.01, post-treatment, with baseline.

^#^P < 0.05, ^##^P < 0.01, comparisons between the IMFG and AG groups at corresponding time points.

^+^P < 0.05, ^++^P < 0.01, comparisons between the IMFG and CG groups at corresponding time points.

^$^P < 0.05, ^$$^P < 0.01, comparisons between the AG and CG groups at corresponding time points.

In the analysis of the LF variable, the IMFG had higher values than the AG and CG after performing the treatment (*P* < 0.01). Within the IMFG analysis, there was an increased between baseline and post-treatment of 31.61 ± 12.35 n.u. (*p* < 0.01; ES = 0.71; 95% CI of the difference = 24.68 to 38.54). In contrast, the AG showed a decreased between baseline and post-treatment of −19.15 ± 4.47 n.u. (*p* < 0.01; ES = 1.36; 95% CI of the difference = −26.36 to −11.18).

In the analysis of the HF variable, the IMFG had lower values than the AG and CG after performing the treatment (*P* < 0.01). Within the IMFG analysis, there was an increased between baseline and post-treatment of −31.45 ± 12.16 n.u. (*p* < 0.01; ES = 2.80; 95% CI of the difference = −38.30 to −24.60). In contrast, the AG showed an increased between baseline and post-treatment of −19.15 ± 4.47 n.u. (*p* < 0.01; ES = 1.23; 95% CI of the difference = 12.30 to 26.00).

## 4 Discussion

The results of this research suggest an increase in sympathetic activity at the expense of parasympathetic activity, coupled with a decrease in the strength of inspiratory musculature in the IMFG, as well as an increase in parasympathetic activity and a decrease in sympathetic activity in the AG.

The autonomic nervous system (ANS) exerts significant influence, both at an organic and physiological level, in the proper functioning of the human body. Through the ANS, specifically through its two main branches (sympathetic and parasympathetic), control of metabolic and thermoregulatory demands arising from exposure to different stimuli is carried out, by adjustments made at the cardiac, respiratory, and blood flow levels, thanks to the regulation of the function of multiple structures through their innervation in glands, cardiac muscle, or smooth muscle, among others. Although cardiac automatisms are governed by an intrinsic system, heart rate is under the influence of the ANS. On one hand, the parasympathetic system, responsible for decreasing the average heart rate, as well as increasing its variability through the release of Acetylcholine, producing a slower diastolic depolarization (Wehrwein et al., 2016; Immanuel et al., 2023). On the other hand, the sympathetic system will be responsible for increasing that heart rate and decreasing its variability, mediated by the release of adrenaline and noradrenaline, producing a faster diastolic depolarization (Wehrwein et al., 2016). Furthermore, higher levels of sympathetic activation are associated with a greater risk of cardiovascular diseases (Greiser et al., 2009) well as a detriment to cognitive function (Grassler et al., 2021), while higher levels of parasympathetic activation are associated, among other things, with improved tissue regeneration (Davis and Dailey, 2019). There is evidence regarding the lower parasympathetic activity developed in older adults, in favor of greater sympathetic activity (Arantes et al., 2022).

As previously mentioned, HRV is an intrinsic characteristic of heart rate defined as the variability between cardiac pulses during a certain period, which also closely relates to the sympathetic-parasympathetic balance of the ANS (Tiwari et al., 2021; Cygankiewicz and Zareba, 2013). Specifically, measures such as HF or RMSSD are associated with a greater presence of the parasympathetic system, while LF is more related to sympathetic predominance (Caruso et al., 2016). Based on the results obtained in our study, an increase in sympathetic activity, as well as a decrease in parasympathetic activity, is observed in the IMFG. These results are consistent with those found by Welch et al. (2018) who conducted a protocol similar to this study, showing an increase in heart rate and blood pressure in the group subjected to diaphragmatic fatigue. Previous studies have demonstrated how the muscle metaboreflex produced by static exercises induces heart rate regulation through sympathetic activation (Iellamo et al., 1999). Regarding the AG, our results suggest an increase in parasympathetic activity and a decrease in sympathetic activity. These results are consistent with those shown by Rodrigues et al., where participants undergoing a single session of inspiratory muscle loading training showed increases in parasympathetic activity mediated by cardiorespiratory interactions resulting from changes in breathing duration and volume as well as changes in intrathoracic pressure (Rodrigues et al., 2021). However, these results obtained in our study should be interpreted with caution, as they are acute interventions with short-term effects, since current literature demonstrates that to achieve HRV adaptations through inspiratory loading work, training periods of 4–8 weeks are needed (Ferreira et al., 2013).

Regarding inspiratory musculature, in the case of older adults, there is a decrease in strength and contraction speed due, among other factors, to changes in thoracic cage morphology, increases in residual lung volume, or sarcopenia (Ohara et al., 2018), presenting greater fatigue of this musculature and a greater tendency to respiratory metaboreflex (Smith et al., 2017). This fatigue of the inspiratory musculature, through afferent stimuli by III and IV fibers, produces an increase in efferent sympathetic activity, resulting in a peripheral vasoconstrictor response, which is further accentuated in older adults (Smith et al., 2017; Dempsey et al., 2006). Our results show a decrease in inspiratory muscle strength in the IMFG, consistent with previous publications (Welch et al., 2018; Smith et al., 2017). This decrease in inspiratory strength was evaluated through variables such as MIP, which is also correlated with quality of life in this population (Roldan et al., 2021) and ultrasound variables at the diaphragmatic level such as thickness, mobility, and diaphragmatic contraction velocity, correlated with transdiaphragmatic pressure (Koco et al., 2021), inspiratory muscle strength (Cardenas et al., 2018) and contractile efficiency of this musculature (Sarwal et al., 2013).

This study has several limitations. On one hand, as previously shown, the results should be interpreted with caution since they demonstrate the effects of an intervention immediately after it was performed, without knowing how long these effects may last. On the other hand, inspiratory muscle fatigue was evaluated using valid methods, but based on available literature, it is known that greater objectivity is achieved in ensuring fatigue through stimulation using evoked potentials of the phrenic nerve or transdiaphragmatic pressure measurements. Additionally, although correct, the sample size of the study is small, and the sample comes from a single nursing home. Based on the data obtained, future studies, with a larger sample size, could conduct different measurements over time to assess how long the effects of such intervention may last, as well as perform such evaluation in different clinical populations such as cardiac patients, COPD patients, or patients with neurological diseases.

## 5 Conclusion

In conclusion, inspiratory muscle fatigue appears to have a negative effect on heart rate variability and respiratory muscle strength, resulting in increased sympathetic activity and decreased parasympathetic activity. Conversely, activation of the inspiratory muscles suggests an enhancement of parasympathetic activity and a reduction in sympathetic activity, along with improvement in respiratory muscle strength.

## Data Availability

The raw data supporting the conclusions of this article will be made available by the authors, without undue reservation.
